# Lactate dehydrogenase A promotes the invasion and proliferation of pituitary adenoma

**DOI:** 10.1038/s41598-017-04366-5

**Published:** 2017-07-05

**Authors:** Jiayin An, Yin Zhang, Jiaojiang He, Zhenle Zang, Zheng Zhou, Xiangdong Pei, Xin Zheng, Weihua Zhang, Hui Yang, Song Li

**Affiliations:** 1 Multidisciplinary center for pituitary adenomas of Chongqing, Department of Neurosurgery, Xinqiao Hospital, Third Military Medical University, Chongqing, China; 2 Department of Neurosurgery, Lanzhou General Hospital of Chinese People’s Liberation Army, Lanzhou, China; 3 Department of Biology and Biochemistry, College of Natural Sciences and Mathematics, University of Houston, Texas, USA

## Abstract

Lactate dehydrogenase A (LDHA) has been reported to be involved in the initiation and progression of tumors. However, the potential role of LDHA in pituitary adenoma (PA) remains unknown. In this study, we showed that the expression levels of LDHA mRNA and protein were significantly elevated in invasive PA samples, and positively correlated with higher Ki-67 index. Overexpression of LDHA in a PA cell line (GH3) promoted glucose uptake through the upregulation of glucose transporter-1 (Glut1), lactate secretion and induced cellular invasion by upregulation of matrix metalloproteinase2 (MMP2). LDHA also promoted GH3 cell proliferation through induction of cell cycle progression via activation of the Akt-GSK-3β-cyclinD1 pathway. Accordingly, oxamate-induced inhibition of LDHA suppressed glucose uptake, lactate secretion, invasion and proliferation in GH3 cells via down regulation of Glut1 and MMP2 expression and inhibition of the Akt-GSK-3β-cyclinD1 pathway. Moreover, oxamate induced GH3 cell apoptosis by increasing mitochondrial reactive oxygen species (ROS) generation. *In vivo*, LDHA overexpression promoted tumor growth, and oxamate delayed tumor growth. In primary PA cell cultures, oxamate also effectively suppressed invasion and proliferation. Our data indicate that LDHA is involved in promoting the progression of PA, and oxamate might be a promising therapeutic agent for the treatment of PA.

## Introduction

Human pituitary adenomas (PA) are generally considered benign tumors that account for 10–15% of primary intracranial neoplasms^1^. Despite their histologically benign nature, a subset of PA can invade the surrounding tissues, including the dura, bones, and sinuses. These adenomas are called “invasive PA”^2–4^. Invasive PA have significantly higher proliferation rates than noninvasive adenomas and are associated with poor prognosis^5^. The treatments for PA include surgery, medical management and radiotherapy. However, some cases of PA respond poorly to these approaches due to their size, invasiveness and rapid growth. Previous studies have reported that genetic changes^6^, epigenetic events^7^, inflammatory mediators^8^ and factors involved in proliferation^9^ are highly associated with the progression of PA. However, the underlying mechanism that governs the invasive behavior of PA remains largely unknown. Thus, clarification of the factors responsible for the aggressive behavior of PA is crucial for developing novel treatment strategies and evaluating prognosis in patients with PA.

Invasive PA is regarded as an intermediate stage in tumor development toward carcinoma^10^. Despite their histologically benign status, invasive PA have a tendency toward malignant biological behavior. It is noticed long before that malignant tumor cells have enhanced glycolysis. Cells have higher uptake of glucose and more lactate production, even in oxygen-rich conditions. This phenomenon is known as the “Warburg effect”^11, 12^.

Lactate dehydrogenase A (LDHA), which converts pyruvate to lactate, is one of the key enzymes involved in glycolysis. Many malignant tumors have higher LDHA levels compared to normal tissues^13^. Multiple studies have shown that LDHA plays an important role in the growth, invasion and metastasis of malignant tumors^14–16^. However, the link between LDHA and the aggressive behavior of PA is unknown.

In this study, we evaluated the expression of LDHA in PA tissues and analyzed LDHA function in the GH3 PA cell line both *in vitro* and *in vivo*. Furthermore, we explored the inhibitory effect and underlying mechanisms of oxamate, a competitive LDHA inhibitor^17^, on GH3 cells and primary culture cells. Our findings support the use of novel therapeutic strategies for patients with PA, particularly those with invasive PA.

## Results

### Increased expression of LDHA in invasive PA samples positively correlated with a higher Ki-67 index

**Figure 1:**
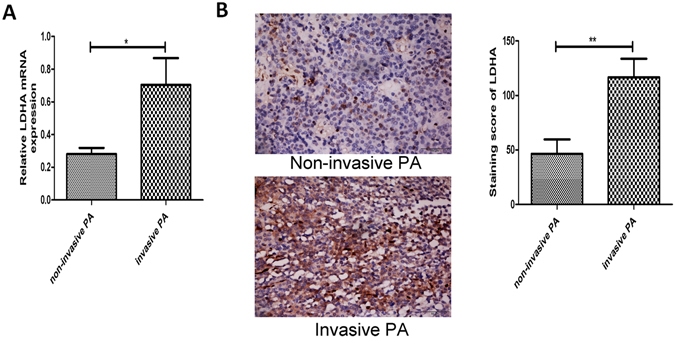
Expression of LDHA in human PA samples. (**A**) The expression of LDHA mRNA in human PA tissures were detected by qRT-PCR. (**B**) The expression of LDHA protein in human PA samples were examined by IHC staining. Original magnification, ×400. *P < 0.05; **P < 0.01.

Both LDHA mRNA and protein were detected in PA samples. LDHA mRNA was highly expressed in the invasive PA samples (P < 0.05, Fig. 1A). LDHA protein was mainly localized to the cytoplasm. Quantification of LDHA levels by semi-quantitative optical analysis was conducted as described in our previous study^18^, and LDHA expression was upregulated in invasive PA compared to noninvasive PA (P < 0.05, Fig. 1B). Ki-67 is a marker of cell proliferation, and a Ki-67 index of 3% is one of the criteria for identification of atypical PA during pathological examination. Our data also indicated that the Ki-67 index positively correlated with LDHA expression (Table 1). These results suggested that LDHA may be an important regulator of the invasion and proliferation of PA cells.

**Table 1:** The relationship between LDHA and Ki-67 index in pituitary adenomas (n = 40).

Ki-67	Total	LDHA		
		−	+	P value
(−)	19	10	9	0.010
(+)	21	3	18	

The immunostaining score of LDHA was scored as negative (−) to indicate staining score with less than 30, positive (+) to indicate score with more than 30.

### LDHA promoted glucose uptake, lactate secretion and invasion in PA cells

**Figure 2:**
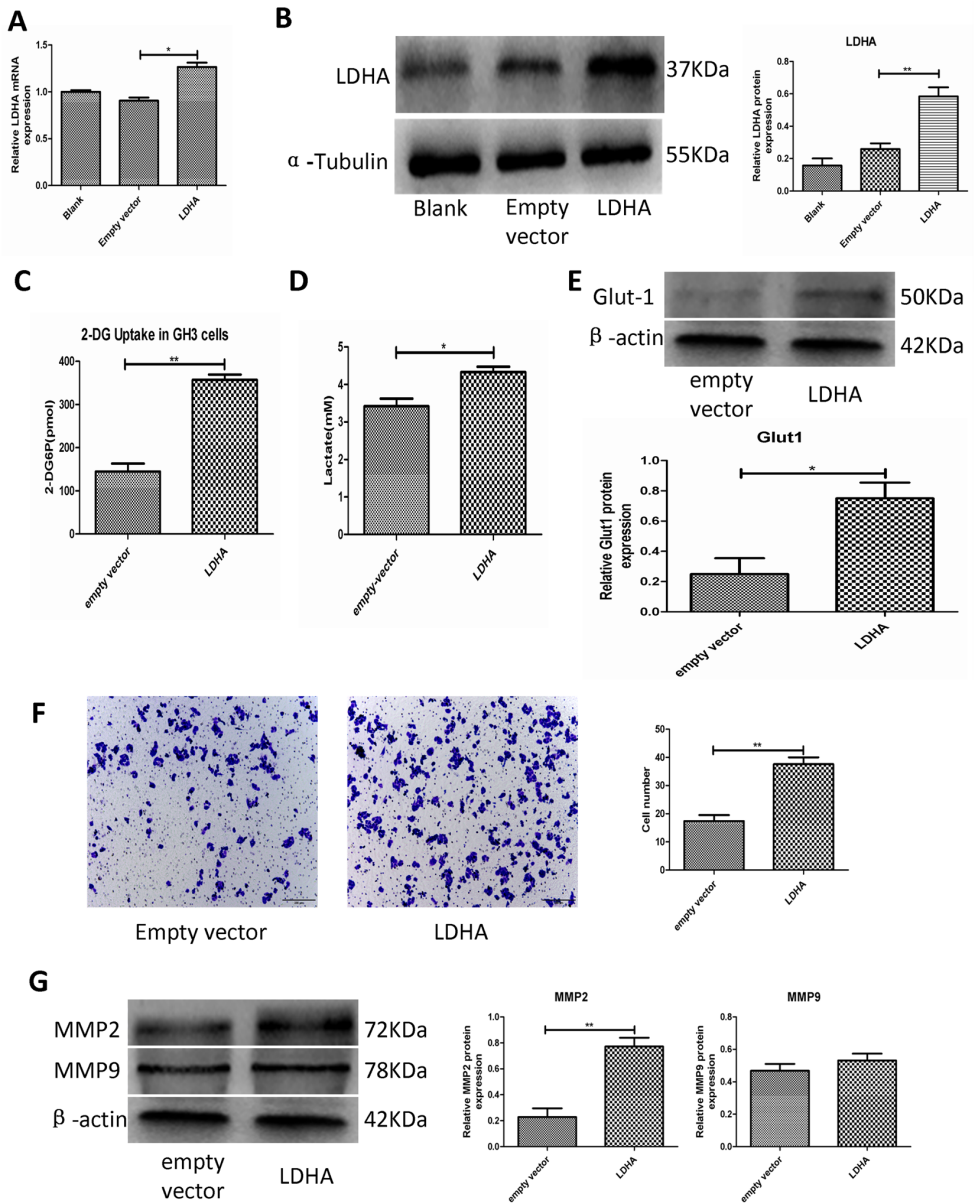
LDHA promote glucose uptake, lactate secretion and invasion in GH3 cells. (**A** and **B**) Measured LDHA mRNA and protein levels in three groups of GH3 cells (blank, transfected with empty vector or LDHA over expression vector) by RT-qPCR and Western blot (n = 3, ±SEM). (**C**) The histogram represents glucose uptake of GH3 cells in two groups of GH3 cells transfected with empty vector or LDHA over expression vector (n = 3, ±SEM). (**D**) The histogram represents lactate proudction of GH3 cells in aboved two groups (n = 3, ±SEM). (**E**) Protein expression level of Glut-1 was examined by Western blot in aboved two groups (n = 3, ±SEM). (**F**) Cell invasion of the aboved two groups was evaluated by transwell assay, Original magnification, ×100 (n = 5, ±SEM). G. Protein expression levels of MMP-2 and MMP-9 were examined by Western blot in aboved two groups (n = 3, ±SEM). *P < 0.05; **P < 0.01.

Because LDHA is a key glycolysis enzyme, we investigated the role of LDHA in the regulation of glucose uptake and lactate secretion which are two key indicators of glycolysis in PA cells. Forced expression of LDHA in GH3 cells by lentiviral vector led to stable upregulation of LDHA (Fig. 2A,B and Fig. S1). We found that LDHA overexpression increased glucose uptake (Fig. 2C) and lactate production in PA cells (Fig. 2D). In addition, glycolytic transporter 1 (Glut1) expression significantly increased after induction of LDHA in GH3 cells (Fig. 2E). Furthermore, we evaluated the effect of LDHA overexpression on GH3 cell invasion. Elevated expression of LDHA promoted cell invasion (Fig. 2F). We also found that LDHA induced the expression of matrix metalloproteinase 2 (MMP2), an important regulatory protein involved in invasion, but not matrix metalloproteinase 9 (MMP9) (Fig. 2G).

### LDHA promoted GH3 cell proliferation by inducing cell cycle progression via the Akt-GSK-3β-cyclinD1 pathway

**Figure 3:**
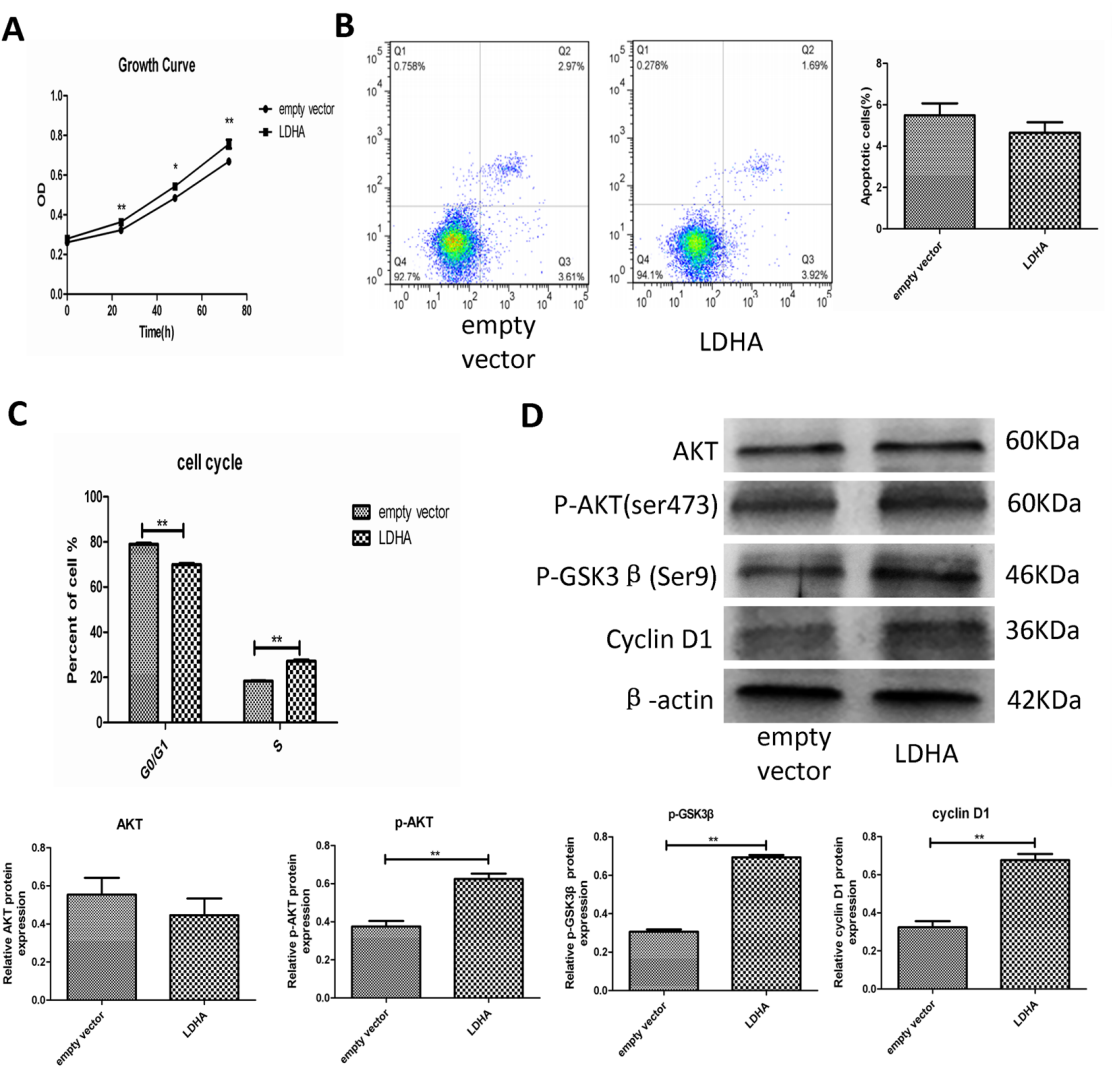
GH3 cells cycle progression promoted by LDHA is mediated by Akt-GSK-3β-cyclinD1 signal pathway. (**A**) Control and LDHA over expression GH3 cells proliferation were tested by CCK-8 assay (n = 6, ±SEM). (**B**) Cell apoptosis of the aboved two groups were measured by flow cytometry (n = 3, ±SEM). (**C**) Cell cycle of control and LDHA over expression cells were analyzed using flow cytometry (n = 3, ±SEM). (**D**) Protein expression levels of Akt, p-Akt, p-GSK3β and CyclinD1 were examined by Western blot (n = 3, ±SEM). *P < 0.05; **P < 0.01.

Because LDHA expression was positively correlated with the Ki-67 index, we explored the effect of LDHA on GH3 cell proliferation. We found that overexpression of LDHA promoted GH3 cell proliferation (Fig. 3A). To investigate the mechanism underlying the effect of LDHA overexpression on the growth and proliferation of PA cells, we determined whether forced expression of LDHA affected apoptosis and the cell cycle in GH3 cells. We used flow cytometry in GH3 cells transfected with either empty vector or an LDHA vector. LDHA overexpression did not significantly alter the percentage of apoptotic cells (Fig. 3B), whereas LDHA overexpression significantly induced S phase transition (Fig. 3C and Fig. S2). We examined the expression of cyclin D1, which is the main cyclin controlling the G1/S phase transition. LDHA overexpression induced increased expression of cyclin D1 (Fig. 3D). The Akt-GSK-3β signaling pathway has a well-characterized, important role in the regulation of cyclin D1 expression^19^. Akt activation inhibits GSK-3β function by phosphorylating GSK-3β at Ser9, leading to stabilization and upregulation of cyclin D1. Therefore, we assessed whether the Akt-GSK-3β-Cyclin D1 signaling pathway was involved in the LDHA-induced increase of cyclin D1 in GH3. We found that forced expression of LDHA promoted phosphorylation of Akt. Consistent with the activation of Akt, phosphorylation of GSK-3β was also increased(Fig. 3D). These findings suggested that LDHA promotion of GH3 cell cycle progression may be mediated by the Akt-GSK-3β-cyclinD1 signaling axis.

### Oxamate inhibited PA cell glucose uptake, lactate secretion and invasion

**Figure 4:**
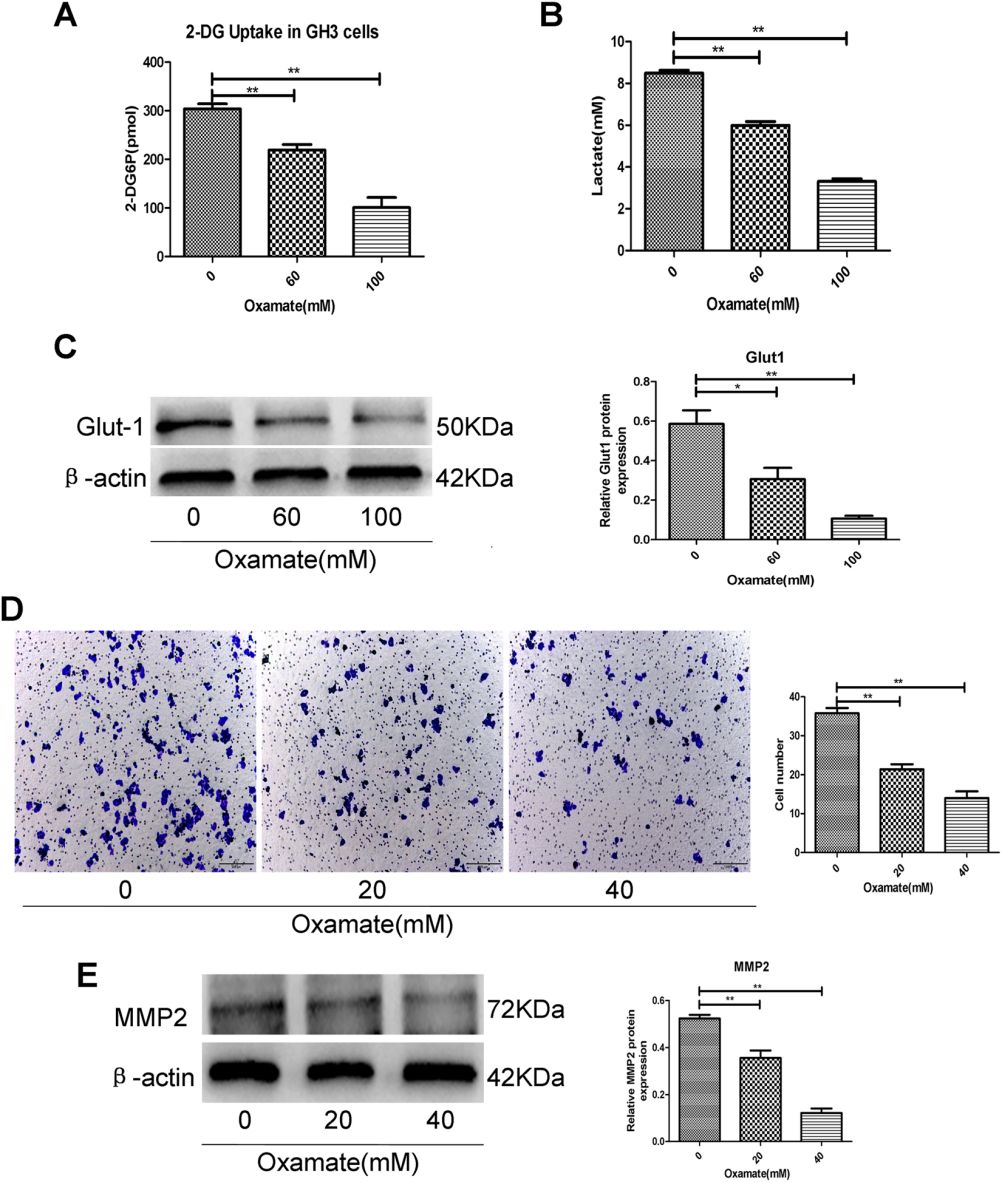
Inhibiting LDHA by oxamate decreased cell glucose uptake, lactate secretion and invasion. (**A**) The histogram represents glucose uptake of GH3 cells were exposed to 0, 60, and 100 mM oxamate for 48 h (n = 3, ±SEM). (**B**) The histogram represents lactate proudction of GH3 cells in aboved groups (n = 3, ±SEM). (**C**) Protein expression level of Glut-1 was examined by Western blot in aboved groups (n = 3, ±SEM). (**D**) GH3 cells were exposed to 0, 20, and 40 mM oxamate for 48 h, and then cell invasion were evaluated by transwell assay, Original magnification, ×100 (n = 5, ±SEM). (**E**) GH3 cells were exposed to 0, 20, and 40 mM oxamate for 48 h and then protein expression levels of MMP-2 were examined by Western blot (n = 3, ±SEM). *P < 0.05; **P < 0.01.

Oxamate is a pyruvate analogue and competitive inhibitor of LDHA. We further investigated the effect of oxamate-induced LDHA inhibition on glucose uptake, lactate secretion and invasion of GH3 cells. By suppressing the activity of LDHA, oxamate significantly inhibited glucose uptake (Fig. 4A), lactate production (Fig. 4B) and Glut1 expression (Fig. 4C) in GH3 cells in a dose-dependent manner. After GH3 cells were treated with different concentrations of oxamate, we found that invasion and the expression of invasion regulatory protein (MMP2) were also reduced in a dose-dependent manner (Fig. 4D,E).

### Oxamate suppressed GH3 cell proliferation by inducing cell cycle arrest via the Akt-GSK-3β-cyclinD1 pathway

**Figure 5:**
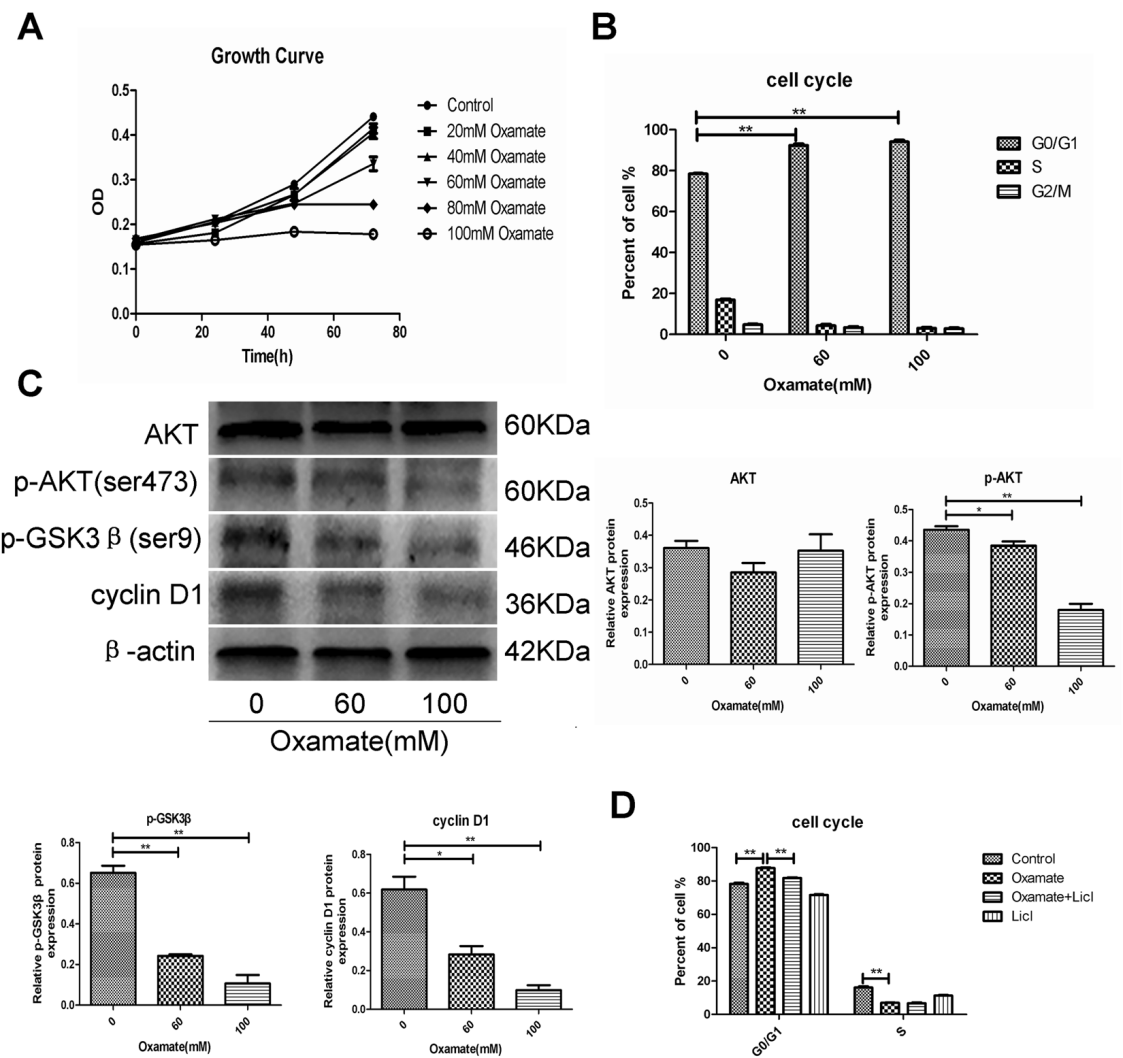
Akt-GSK-3β-cyclinD1 signal pathway mediates Cell cycle arrested by oxamate in GH3 cells. (**A**) GH3 cells were incubated with different concentrations of oxamate for 0, 24, 48 and 72 h, and cell proliferation were tested by CCK-8 assay (n = 3, ±SEM). (**B**) GH3 cells were exposed to 0, 60, and 100 mM oxamate for 48 h, and then cell cycle were analyzed using flow cytometry (n = 3, ±SEM). (**C**) GH3 cells were exposed to 0, 60, and 100 mM oxamate for 48 h, and protein expression levels of Akt, p-Akt, p-GSK3β and CyclinD1 were examined by Western blot (n = 3, ±SEM). (**D**) GH3 cells were pretreated with or without Licl and then treated with oxamate. Cell cycle were analyzed using flow cytometry (n = 3, ±SEM). *P < 0.05; **P < 0.01.

Because LDHA significantly promoted PA cell proliferation by inducing cell cycle progression, we conducted CCK-8 assays to investigate the role of oxamate in the proliferation of GH3 cells. Our study revealed that oxamate inhibited GH3 proliferation in a dose-dependent manner (Fig. 5A). We also investigated alterations in cell cycle distribution after oxamate treatment. We found that oxamate arrested cell cycle progression at G0/G1 in GH3 cells in a dose-dependent manner (Fig. 5B and Fig. S3). Additionally, oxamate suppressed phosphorylation of Akt and GSK-3β. Consistent with GSK-3β activation, cyclin D1 expression was also reduced (Fig. 5C). These findings suggested that oxamate may downregulate cyclin D1 expression by suppressing the activation of Akt and activating GSK-3β. To further examine the role of the Akt-GSK-3β-cyclin D1 signaling pathway in the G0/G1 arrest induced by oxamate, we treated GH3 cells with both LiCl, a GSK-3β inhibitor, and oxamate. Our results demonstrated that LiCl effectively reversed the G0/G1 arrest induced by oxamate (Fig. 5D and Fig. S4). Altogether, our findings indicated that oxamate inhibited the Akt-GSK-3β pathway, which led to the degradation of cyclin D1 and eventually cell cycle arrest at the G0/G1 phase.

### Oxamate induced apoptosis via the reactive oxygen species (ROS)-mediated mitochondrial apoptotic pathway

**Figure 6:**
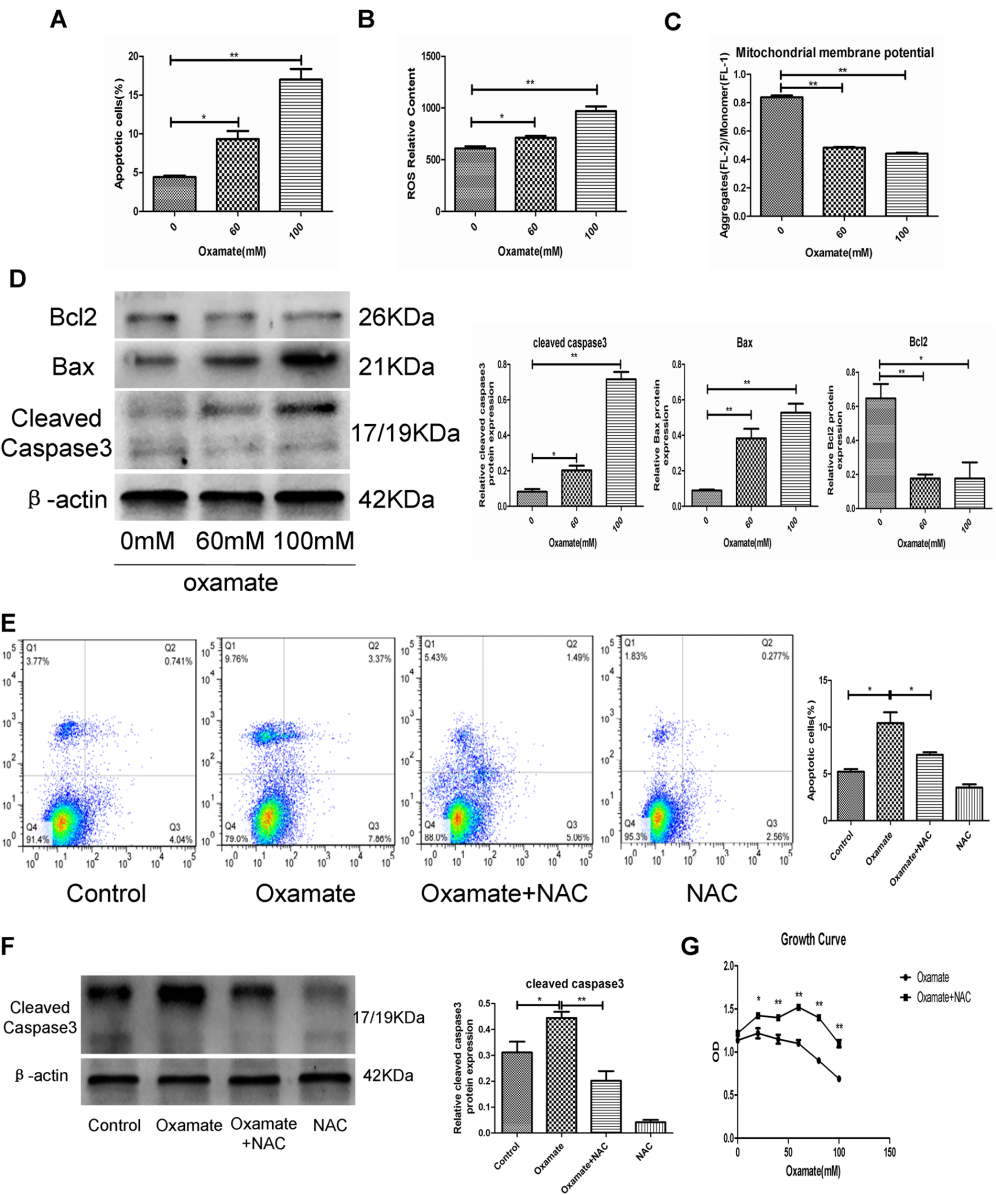
ROS triggered mitochondrial apoptosis pathway of GH3 cells after treatment with oxamate. (**A**) GH3 cells were exposed to 0, 60, and 100 mM oxamate for 48 h and cell apoptosis were measured by flow cytometry (n = 3, ±SEM). (**B**) Intracellular ROS levels of different treatment groups were determined by DCFH-DA fluorescence (n = 3, ±SEM). (**C**) Mitochondrial membrane potential were measured in different treatment groups by JC-1 staining (n = 3, ±SEM). (**D**) Protein expression levels of Bcl2, Bax and Cleaved caspase3 were examined by Western blot (n = 3, ±SEM). (**E** and **F**) GH3 cells were pretreated with or without NAC and then treated with oxamate. Apoptosis were measured by flow cytometry and cleaved-caspase3 were examined by Western blot (n = 3, ±SEM). (**G**) GH3 cells were incubated with different concentrations of oxamate combined with 10 mM NAC or without for 48 h, and cell proliferation were tested by CCK-8 assay (n = 6, ±SEM). *P < 0.05; **P < 0.01.

We also assessed the impact of oxamate on GH3 cell apoptosis. GH3 cells were treated with different concentrations of oxamate, and then the percentage of apoptotic cells was quantified by flow cytometry. The percentage of apoptotic cells was significantly increased by oxamate in a dose-dependent manner (Fig. 6A and Fig. S5A). We also performed DCFH-DA and JC-1 staining to detect changes in intracellular ROS accumulation and mitochondrial membrane potential in GH3 cells after oxamate treatment. We found that oxamate enhanced ROS levels and reduced mitochondrial membrane potential in a dose-dependent manner (Fig. 6B,C and Fig. S5B,C). Bcl-2 and Bax are two important regulators of the mitochondrial apoptotic pathway^20^. Thus, we measured the expression of several mitochondrial apoptotic pathway-related proteins, including Bcl-2, Bax and cleaved caspase-3, using western blot analysis. Our results showed that expression of the anti-apoptotic protein Bcl-2 was decreased by oxamate in a dose-dependent manner. Conversely, expression of the pro-apoptotic proteins Bax and cleaved caspase-3 were significantly increased (Fig. 6D). To further clarify the role of ROS in oxamate-induced apoptosis, we treated GH3 cells with both N-acetylcysteine (NAC), a specific ROS scavenger, and oxamate. Our results showed that NAC significantly reversed apoptosis and suppressed the expression of cleaved caspase-3 induced by oxamate in GH3 cells (Fig. 6E,F). In addition, NAC also effectively reversed the inhibitory effect of oxamate on GH3 cell growth (Fig. 6G). These findings indicated that ROS generation mediated oxamate-induced PA cell apoptosis through the mitochondrial pathway.

### LDHA regulated GH3 cell growth *in vivo*

**Figure 7:**
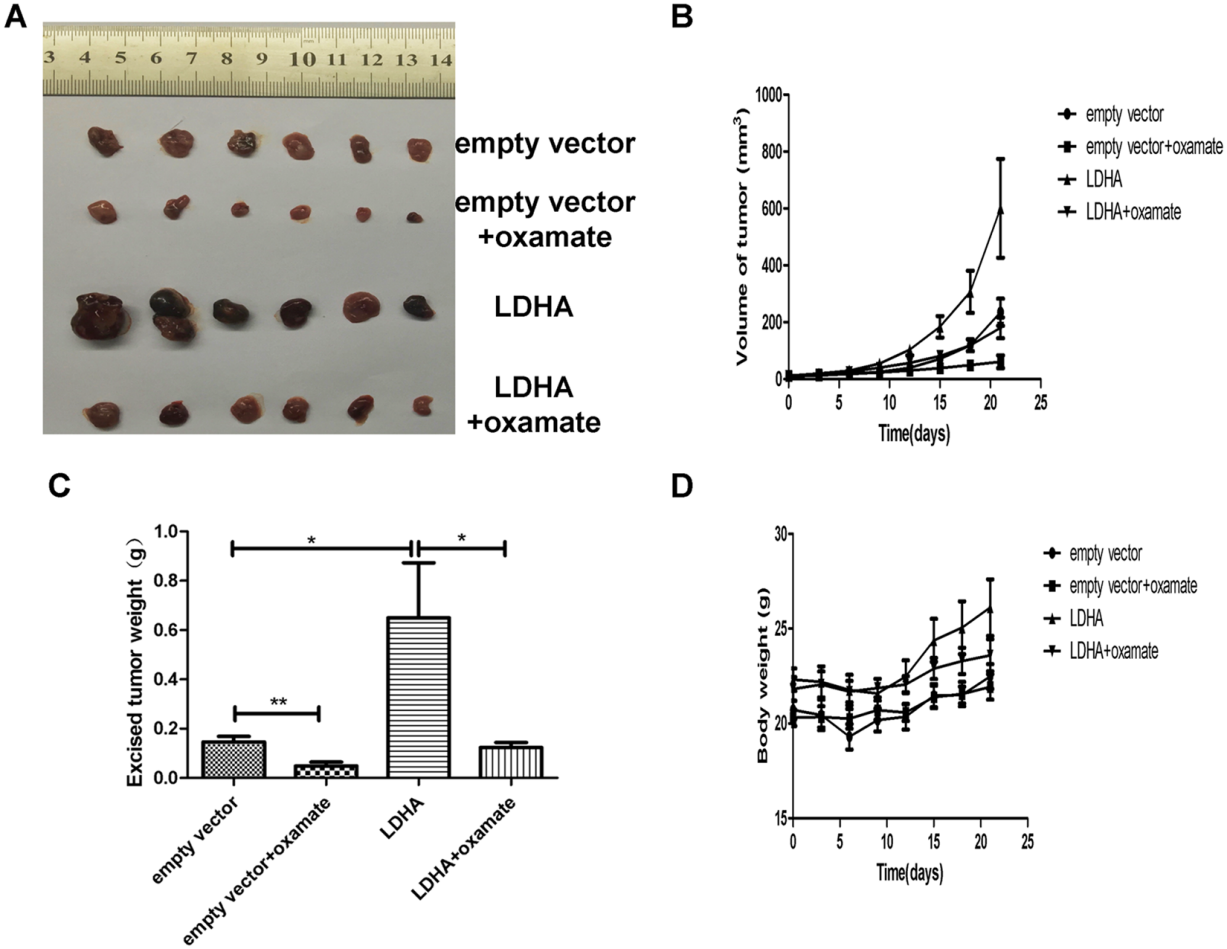
LDHA regulated growth of GH3 cells *in vivo*. (**A**) GH3 cells of transfected with empty vector or LDHA were injected subcutaneously in nude mice, excised tumors of different groups are shown. (**B**) Growth curve showed the tumor volume change of mice after administration of vehicle or oxamate. (**C**) The weight of the excised tumors in each group. (**D**) Body weight of mice in each group. *P < 0.05; **P < 0.01.

To further evaluate the effects of LDHA on GH3 cells and the antitumor potential of oxamate *in vivo*, we generated PA xenograft model by subcutaneous injection of either GH3 empty vector control or cells overexpressing LDHA into nude mice. The mice were randomly divided into four groups (empty vector, empty vector + oxamate, LDHA, LDHA + oxamate). After nine days of injections, all mice were administered vehicle or oxamate each day for 3 weeks. We found that LDHA overexpression significantly promoted tumor growth, while oxamate significantly delayed tumor growth *in vivo* (Fig. 7A,B,C). Additionally, oxamate did not significantly alter mice body weight (Fig. 7D).

### Oxamate inhibited the invasion and proliferation of human PA cells derived from patients

**Figure 8:**
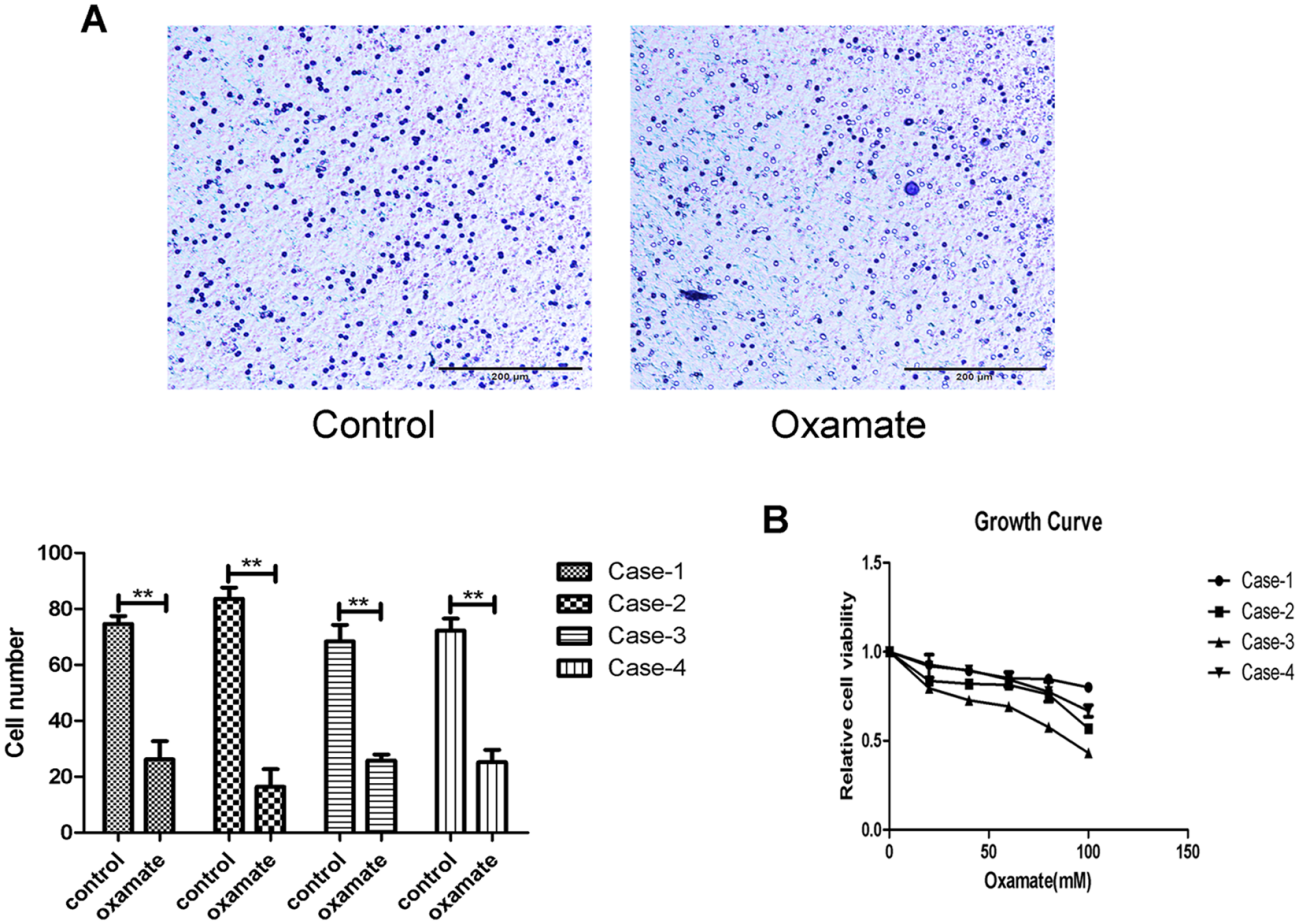
Oxamate suppressed cellular invasion and proliferation in primary cultures of human PA cells. (**A**) Detection of the primary cells invasion after untreated or treated with oxamate (40 mM) for 48 hours by transwell assay, Original magnification, ×100 (n = 5, ±SEM). (**B**) The effect of oxamate on proliferation of the primary tumor cells, after 72 hours of exposure (n = 3, ±SEM). **P < 0.01.

To further investigate the effect of LDHA on the invasion and proliferation of primary PA cells, we cultured primary cells derived from 4 human invasive pituitary adenomas after transsphenoidal resection. Freshly isolated primary PA cells were treated with oxamate and assessed at 48 hours. Similar to results in the GH3 cell line, oxamate inhibited PA cell invasion in all cultures derived from patients (Fig. 8A). Furthermore, significant suppression of PA cell growth was detected in all cultures after treatment with different concentrations of oxamate for 72 hours (Fig. 8B). These data further verified the inhibitory effects of oxamate on PA.

## Discussion

Little attention has been paid to the potential role of cellular metabolic enzymes on the progression of PA. For the first time, we demonstrated that elevated LDHA was correlated with the invasiveness and proliferation of human PA samples and confirmed that overexpression of LDHA promoted PA cell invasion and proliferation both *in vitro* and *in vivo*. We also investigated the inhibitory effect of LDHA inhibition by oxamate and the mechanisms of oxamate treatment in PA cells both *in vitro* and *in vivo*.

The “Warburg effect” is the most important metabolic changes in malignant tumors. Aerobic glycolysis is known to be involved in the progression of several types of tumors. Increased glycolysis and lactate production in tumor cells promote growth and invasiveness^21–23^. LDHA, a key glycolysis enzyme, catalyzes the conversion of pyruvate to lactate in the final step of glycolysis. Expression of LDHA could be potentially elevated in malignant tumors compared with normal tissues^24^. In our study, we initially found that human invasive PA have higher LDHA levels than noninvasive adenomas. Moreover, high LDHA expression in PA tissue was positively associated with the Ki-67 index, an indicator of cell proliferation. Ki-67 is also used to evaluate the aggressive behavior of PA^25^. Therefore, these results indicate that LDHA may be involved in the progression of PA.

The important role of LDHA in tumor invasion and proliferation has been reported in several types of tumors^26, 27^. In our study, forced expression of LDHA in GH3 cells enhanced glucose uptake, lactate secretion and invasion. Suppression of LDHA by oxamate, which has great potential as a promising antitumor drug^28, 29^, reduced glucose uptake, lactate secretion and invasion in GH3 cells. Several studies have confirmed that MMP2 is closely related to the invasiveness of PA^30, 31^. In addition, LDHA has been shown to be involved in the regulation of MMP2 expression^32^. Our results also revealed that upregulation of LDHA in GH3 cells increased the expression of MMP2. Inhibiting LDHA with oxamate downregulated the expression of MMP2. These data indicated that LDHA promoted the invasion of GH3 cells via upregulation of MMP2.

Furthermore, we found that LDHA induced elevation of GH3 cell proliferation, and oxamate reversed the growth-promoting effect of LDHA both *in vitro* and *in vivo*. Our data also showed that LDHA promoted the PA cell transition into the S phase and upregulated cyclin D1, which is a major regulator of the cell cycle^33^. The Akt-GSK-3β–Cyclin D1 signaling pathway plays a crucial role in the regulation of the cell cycle during the G1/S transition. Akt can inactivate GSK-3β kinase activity via phosphorylation at Ser9, which subsequently reduces cyclin D1 phosphorylation. Increased cyclin D1 drives cells into the S phase^34, 35^. Several studies have reported inhibition of Akt activity, a cardinal node in many signaling pathways, after suppression of LDHA^36, 37^. Consistent with previous studies, our results showed that upregulation of LDHA in PA cells activated the Akt-GSK-3β–Cyclin D1 signaling pathway. Oxamate-induced inhibition of LDHA also arrested GH3 cells at the G0/G1 phase via suppression of the Akt-GSK-3β–Cyclin D1 signaling pathway. Therefore, these results suggested that LDHA may promote GH3 cell proliferation through activation of the Akt-GSK-3β–Cyclin D1 signaling pathway. Importantly, we further explored the inhibitory effects of oxamate on the invasiveness and growth of primary human PA cells and found that primary cultured cells from patients with PA responded to oxamate treatment.

Intriguingly, we also found that oxamate significantly increased GH3 cell apoptosis in a dose-dependent manner. The mitochondrial-mediated pathway is an important mechanism for induction of apoptosis^38^. This pathway is characterized by a decrease in the mitochondrial membrane potential and is regulated by Bcl-2 family proteins^39^. ROS are highly reactive and short-lived small molecules^40^ that are thought to participate in the regulation of a variety of cellular functions, such as proliferation, apoptosis and differentiation. Intracellular ROS are mainly produced in the mitochondria and play a central role in the regulation of mitochondrial-mediated apoptosis. Increased ROS induce depolarization of the mitochondrial membrane, followed by activation of pro-apoptotic molecules in the cell, which eventually leads to apoptosis^41^. Our results revealed that oxamate significantly increased ROS and reduced the mitochondrial membrane potential in GH3 cells. Increased expression levels of the pro-apoptotic proteins Bax and cleaved caspase-3 were induced in oxamate-treated cells; however, oxamate reduced the expression of the anti-apoptotic protein bcl-2. Moreover, NAC, an ROS scavenger, partially reversed oxamate-induced GH3 cell apoptosis. These data suggested that oxamate-induced inhibition of LDHA caused ROS-mediated induction of the intrinsic mitochondrial pathway of apoptosis in PA cells.

In summary, in the present study, we revealed that LDHA expression was dysregulated in invasive PA. In addition, we demonstrated that LDHA plays an important role in invasion as well as PA growth. Inhibiting LDHA with oxamate suppressed invasion and proliferation of PA. Our results suggest that targeting LDHA with molecules such as oxamate has great potential as a promising treatment for patients with PA, especially patients with invasive PA. However, the results of this study must be verified by further clinical investigation.

## Methods

### Patients

A total of 40 human PA samples (13 somatotroph adenoma, 7 prolactinoma, and 20 nonfunctioning adenoma) were obtained from patients who underwent transsphenoidal surgery at the multidisciplinary center for pituitary adenomas of Chongqing of the Xinqiao Hospital. The diagnoses of individual tumors were according to clinical and endocrine assessment with additional information provided by pathological evaluation. Tumor invasion was determined based on preoperative radiological investigation using Knosp’s classification^42^ combined with intraoperative findings. Noninvasive adenomas were grade 0–2, while invasive adenomas were grades 3–4 according to the Knosp classification system. Clinical data regarding gender, age, knosp grade, and hormonal type were summarized in Table S1. This study was approved by the Ethics and Clinical Research Committee of Xinqiao Hospital and informed consent was obtained from all patients or their relatives.

### Immunohistochemistry

Tumor tissues were fixed in paraformaldehyde for 24 hours and then embedded in paraflin. The embedded tumor tissues were sectioned at 5 um for subsequent immunohistochemical staining. The staining procedure was carried out according to the standard protocols. LDHA primary antibody (1:400; CST) was used. An olympus microscopy was used to get the images of the different sections.

### Cell culture and transfection

Rat GH3 pituitary adenoma cell line was acquired from American Type Culture Collection (ATCC, Manassas, VA, USA). GH3 cells were maintained in Ham’s F-12 K medium (GIBCO, Carlsbad, CA, USA) containing 2.5% fetal bovine serum (FBS, GIBCO) and 15% horse serum (HS, GIBCO) in a 5% CO_2_ incubator at 37 °C. Lentiviral vectors (obio, Shanghai, China), empty as well as LDHA overexpression, were transfected to GH3 cells at a multiplicity of infection (MOI) of 100 according to the manufacturer’s instructions. Stable colonies were selected by adding puromycin (2 ug/ml) to GH3 transfectants for 48 hours. Transfection efficiencies were verified by real-time PCR and Western blot.

### Primary culture of human PA cells

Four human invasive PA specimens were obtained from patients undergoing transsphenoidal surgery at Xinqiao Hospital in Chongqing, China (Table S2). Fresh tumor tissues were washed by 1X PBS. Then the washed tissues were cut into small pieces. The tissue fragments were digested with Type I Collagenase for two hours at 37 °C. After adding equal amounts of 10% FBS–containing MEM media, cell suspension was filtered through 200 Mo filter. Then cell suspension was centrifuged and washed by PBS two times, cell pellet was resuspended in 10% FBS–containing MEM media. Finally, the primary cells were cultured in a 5% CO_2_-humidified atmosphere at 37 °C.

### Lactic acid detection

Lactic Acid assay kit (NanJing JianCheng Bioengineering Institute) was used to measure the concentration of lactic acid. GH3 cell culture supernatants in different treatment groups were obtained and the levels of lactic acid were measured following the manufacturer’s instructions

### Analysis of reactive oxygen species (ROS)

ROS content was determined by flow cytometry using an ROS detection kit (Beyotime, Nanjing, China). Briefly, GH3 cells were harvested at the indicated time points with relative treatment. The collected cells were incubated with 10 uM DCFH-DA for 20 min at 37 °C. Each sample was assessed by flow cytometry for fluorescence intensity.

### Invasion assay

Transwell chamber (8-um pore; Costar, Bethesda, MD, USA) were precoated with 100 ul Matrigel (Corning, USA) for half an hour at 37 °C. About 5 × 10^4^ trysinized cells were resuspended in 200 ul serum-free medium and placed into the upper chamber. Adding 500 ul F-12 K complete medium to the lower chamber. After incubating with the indicated treatments for 48 hours, invasive cells were fixed and stained with crystal violet staining solution (Beyotime). Cell numbers were counted using microscopy in 5 different fields at a magnifcation of 200×.

### Glucose uptake assay

1500 GH3 cells were seeded into each well of a 96-well plate. Cells were incubated for 24 hours and then starved in 100 ul of serum-free F-12 K medium overnight. Cells were washed 3 times with PBS and then incubated with KRPH/2% BSA buffer for 40 minutes. Then Glucose Uptake Assay Kit (Colorimetric, ab136955) was used to detect glucose uptake in GH3 cells with relative treatment based on the manufacturer’s instructions.

### *In vivo* experiments

For the *in vivo* PA xenograft research, 4-week-old male BALB/cA-nu mice were purchased from Beijing HFK Bioscience Co. Ltd. (Beijing, China). Nude mice were reared in SPF condition. A total of 3 × 10^6^ GH3 cells transfected with empty vector suspended in 100 ul solution (50% PBS and 50% Matrigel) were subcutaneously inoculated into the right flank of twelve mice, and the same amount of GH3 cells transfected with LDHA were also subcutaneously inoculated into the rest of twelve mice. Treatment with oxamate was started 9 days after inoculation of the cells. Then the tumor bearing animals were randomly divided into 4 groups (6 mice/group). The oxamate-treated group (empty vector + oxamate, LDHA + oxamate) received daily intraperitoneal injection of 750 mg/kg oxamate for the next 3 weeks until the mice were killed, while the control group (empty vector, LDHA) received daily intraperitoneal injection of equal volume of PBS only. The mice were monitored daily for any discomfort. And the mice were weighed and tumors volume were also measured every three days. Tumor volume was calculated using the formula V (mm^3^) = [ab^2^]/2, which a is the length and b is the width of the tumor. Xenograft tumours were resected from tumor-bearing nude mice following the final treatment. All of the animal procedures were conducted according to protocols approved by the Institutional Animal Care and Ethics Committee.

### Reverse transcription and qPCR

Total RNA was extracted using Trizol Reagent (Invitrogen, USA). Then, 1 µg of the total RNA was reverse-transcribed to cDNA using a reverse transcription kit (TaKaRa, Dalian, China). SYBR Premix Ex Taq II (TaKaRa, Dalian, China) and a CFX96 Real-time System (Bio-Rad Laboratories, Hercules, CA, USA) were used to carry out qPCR. The relative expression levels were calculated using the 2^−△△ct^ method. The primer sequences used for qPCR were as follows (Table S3).

### Western blot analysis

Cell extracts equal to 40 ug protein were subjected to SDS-PAGE and then transferred onto polyvinylidene difluoride membranes. The membranes were blocked for 2 hours at room temperature with 5% nonfat milk in Tris-buffered saline containing 0.05% Tween 20, incubated with rabbit antibodies against rat Bcl-2 (1:1000; Abcam), Bax (1:1000; Abcam), Caspase-3 (1:1000; CST), LDHA (1:1000; CST), AKT (1:1000; CST), p-AKT(Ser473) (1:1000; CST), p-GSK-3β(Ser9) (1:1000; CST), Cyclin D1 (1:1000; CST), Glut-1 (1:200; Sigma), MMP-2 (1:5000; Abcam), MMP-9 (1:5000; Abcam), β-actin (1:1000; CST). The membranes were further incubated with horseradish peroxidase-conjugated goat anti-rabbit IgG (1:2000; Santa Cruz Biotechnology). The membrane signals were detected by enhanced chemiluminescence.

### Cell proliferation assay

WST-8 Cell Counting Kit-8 (Dojindo, Kumamoto, Japan) was used to measure cell proliferation based on the manufacturer’s instructions.

### Cell cycle analysis

GH3 cells were seeded into each well of a 6-well plate. Then, cells were collected at the indicated time points with relative treatment. The collected cells were fixed in 70% ethanol at 4 °C overnight. Subsequently, the cells were resuspended and stained in 500 ul propidium iodide (PI; 0.05 mg/ml; BD Biosciences Pharmingen) and analyzed by flow cytometry (FACScan; BD Biosciences Pharmingen, San Diego, CA, USA).

### Apoptosis analysis

GH3 cells were seeded into each well of a 6-well plate. Then, cells were collected at the indicated time points with relative treatment. Apoptosis of collected cells were assessed using Annexin V-PE/7-AAD or FITC-Annexin V apoptosis detection kit (BD Biosciences Pharmingen) based on the manufacturer’s instructions. Finally, apoptosis was detected by flow cytometry (FACScan; BD Biosciences Pharmingen, San Diego, CA, USA).

### Analysis of mitochondrial membrane potential

Mitochondrial membrane potential was determined by flow cytometry using the mitochondrial membrane potential assay kit with JC-1(Beyotime, Nanjing, China). Briefly, GH3 cells were harvested at the indicated time points with relative treatment. Then the collected cells were treated according to the manufacturer’s instructions. Each sample was assessed by flow cytometry for red (JC-1 aggregates) and green (JC-1 monomers) fluorescence. JC-1 emits red fluorescence when it enter intact mitochondria to form aggregates. However, on the condition of mitochondrial membrane potential collapsed, JC-1 forms monomer and gives green fluorescence. So, the ratio of aggregates/monomer reflects the change of mitochondrial membrane potential.

### Statistical analysis

The data were expressed as the means ± SEM. The correlations between the levels of LDHA and Ki-67 index of PAs was determined by chi-square test. And two-tailed Student’s t-test was applied to determine statistical significance between two groups. Data analysis were conducted by SPSS for Windows version 13.0 (SPSS Inc, Chicago, IL, USA). P values less than 0.05 were considered significant.

### Ethical standards

All patients had given their informed consent for processing the specimens. The study was approved by the Ethics and Clinical Research Committee of Xinqiao Hospital. Procedures were conducted in accordance with the Declaration of Helsinki.

## Electronic supplementary material

Lactate dehydrogenase A promotes the invasion and proliferation of pituitary adenoma
