# Supplementary material for: Lactate dehydrogenase A promotes the invasion and proliferation of pituitary adenoma
Source: Sci Rep. 2017 Jul 5;7:4734. doi: 10.1038/s41598-017-04366-5 (PMC5498590; doi:10.1038/s41598-017-04366-5)
Supplement: Supplementary file 1 — Lactate dehydrogenase A promotes the invasion and proliferation of pituitary adenoma [file 41598_2017_4366_MOESM1_ESM.pdf]

Lactate dehydrogenase A promotes the invasion and proliferation of pituitary adenoma

Jiayin An[1], Yin Zhang[1], Jiaojiang He[2], Zhenle Zang[1], Zheng Zhou[1], Xiangdong Pei[1], Xin Zheng[1], Weihua Zhang[3], Hui Yang[1] and Song Li[1]

[1] Multidisciplinary center for pituitary adenomas of Chongqing, Department of Neurosurgery, Xinqiao Hospital,Third Military Medical University, Chongqing, China.

[2] Department of Neurosurgery, Lanzhou General Hospital of Chinese People's Liberation Army, Lanzhou, China.

[3] Department of Biology and Biochemistry, College of Natural Sciences and Mathematics, University of Houston, Texas, USA.

Corresponding authors:
Hui Yang;
Multidisciplinary center for pituitary adenomas of Chongqing, Department of Neurosurgery
Xinqiao Hospital; Third Military Medical University
183 Xinqiao Main Street, Shapingba District, Chongqing, China
Phone:+86 023 68755610
Fax: +86 023 65218204
Email:huiyangxinqiao@163.com
or Song Li;
Multidisciplinary center for pituitary adenomas of Chongqing, Department of Neurosurgery
Xinqiao Hospital; Third Military Medical University
183 Xinqiao Main Street, Shapingba District, Chongqing, China
Phone:+86 023 68755610
Fax: +86 023 65218204
Email: dlisong3@163.com.

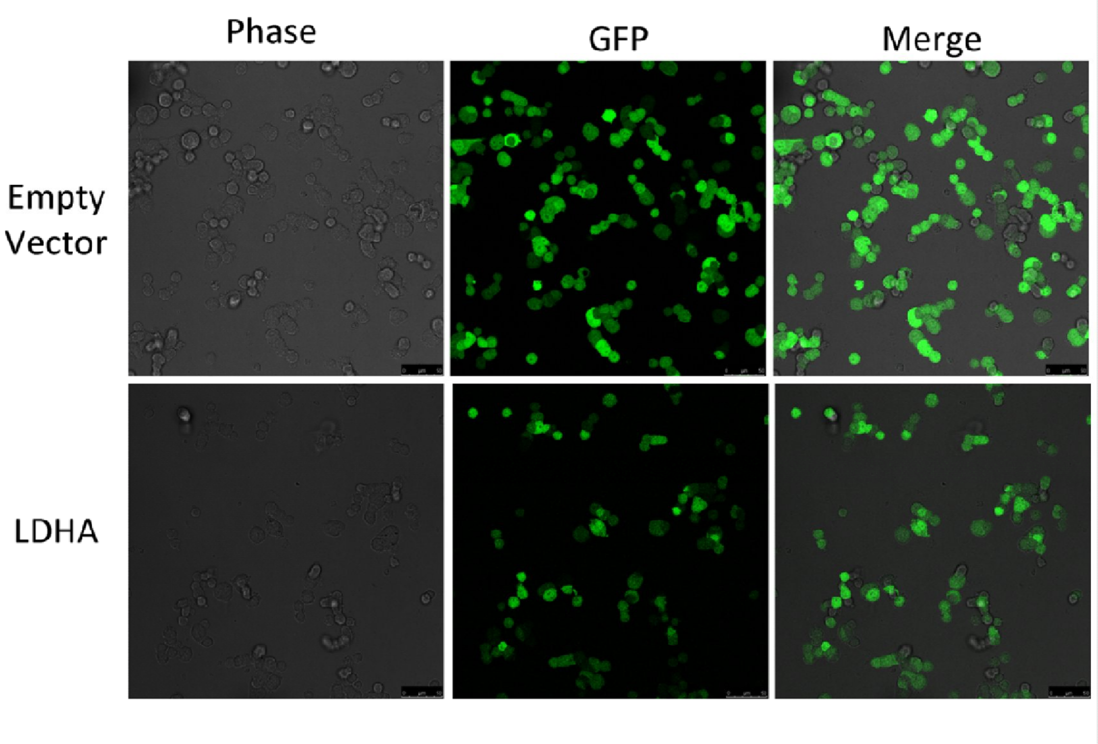

Supplementary Figure S1. Laser scanning confocal microscope examination one week after LDHA transfection with lentivirus, indicating high transfection efficiencies in GH3 cells.

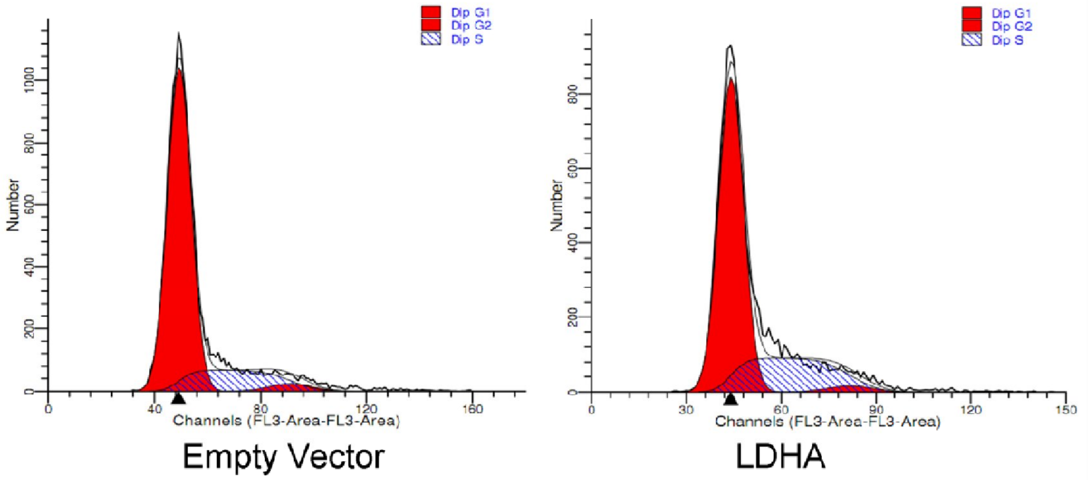

Supplementary Figure S2. Cell cycle distributions of GH3 cells transfected with empty vector or LDHA over expression vector were analyzed using flow cytometry.

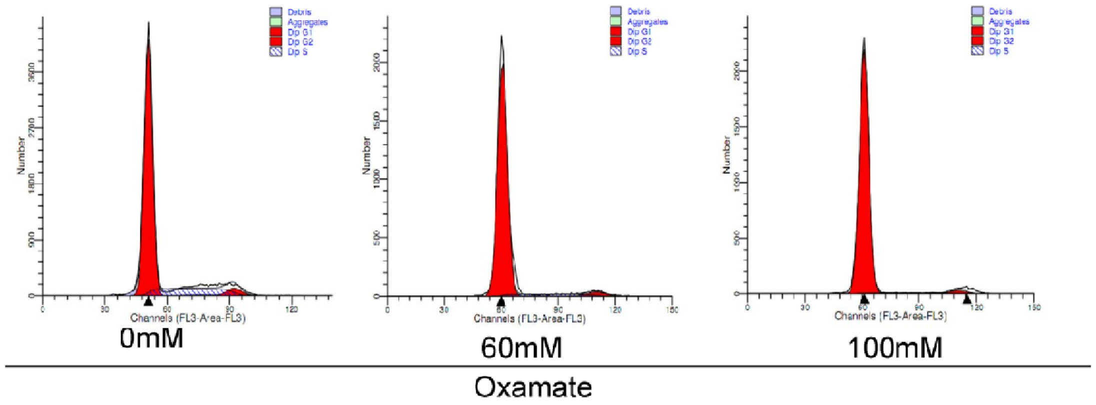

Supplementary Figure S3. GH3 cells were treated with oxamate (0, 60 and 100 mM) for 48 h, and cell cycle distributions were analyzed using flow cytometry.

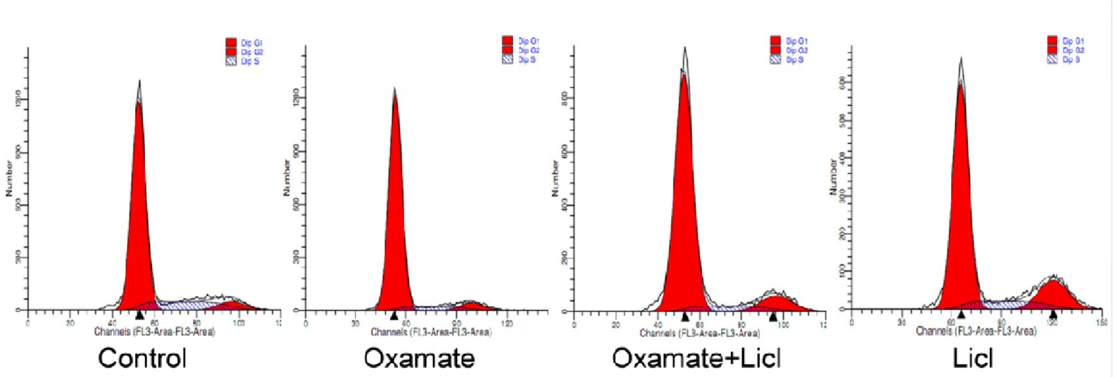

Supplementary Figure S4. GH3 cells were pretreated with or without Licl and then treated with oxamate for 48 h, then cell cycle were analyzed using flow cytometry.

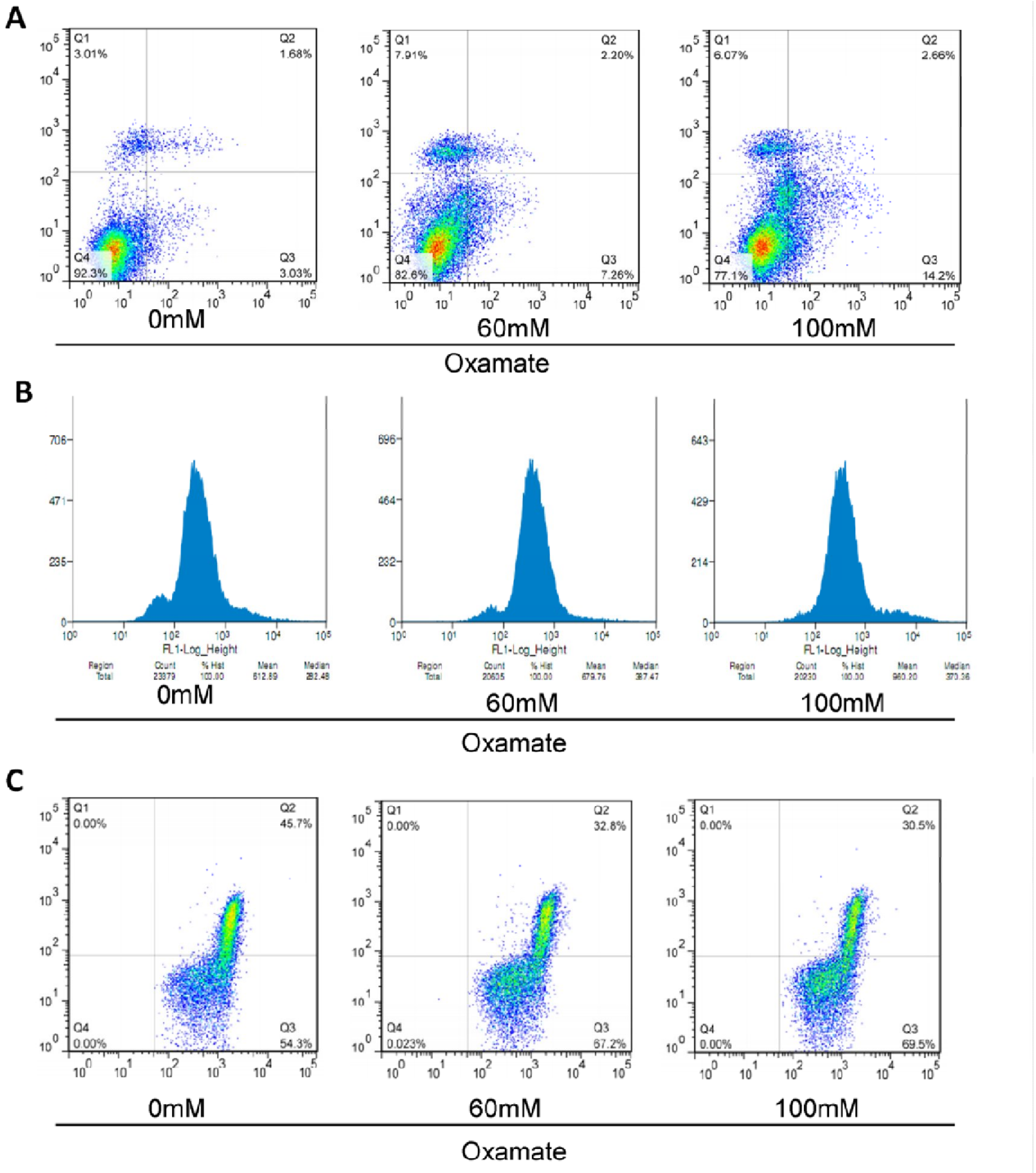

Supplementary Figure S5. A, GH3 cells were treated with oxamate (0, 60 and 100 mM) for 48 h, and cell apoptosis were analyzed using flow cytometry. B, GH3 cells were treated with oxamate (0, 60 and 100 mM) for 48 h, and intracellular ROS levels were analyzed by DCFH-DA fluorescence using flow cytometry. C, GH3 cells were treated with oxamate (0, 60 and 100 mM) for 48 h, mitochondrial membrane potential were measured by JC-1 staining using flow cytometry.

Supplementary Table S1

Patient clinical characteristics.

Case	Age(yrs)	Sex	Knosp grade	Hormonal type	Ki-67
1	52	M	I	PRL	+
2	57	M	II	GH	-
3	38	F	II	NFPA	+
4	35	M	I	PRL	-

**5**	**33**	**F**	**I**	**PRL**	**-**
**6**	**51**	**M**	**II**	**NFPA**	**-**
**7**	**49**	**F**	**II**	**GH**	**-**
**8**	**46**	**F**	**II**	**GH**	**-**
**9**	**48**	**F**	**II**	**GH**	**-**
**10**	**51**	**F**	**I**	**NFPA**	**-**
**11**	**61**	**M**	**I**	**NFPA**	**-**
**12**	**32**	**F**	**II**	**NFPA**	+
**13**	**32**	**M**	**II**	**GH**	**-**
**14**	**61**	**F**	**I**	**NFPA**	**-**
**15**	**45**	**F**	**I**	**NFPA**	**-**
**16**	**51**	**F**	**II**	**NFPA**	+
**17**	**19**	**F**	**I**	**PRL**	+
**18**	**51**	**F**	**I**	**GH**	+
**19**	**45**	**F**	**II**	**GH**	+
**20**	**63**	**M**	**II**	**NFPA**	**-**
**21**	**66**	**F**	**IV**	**NFPA**	**-**
**22**	**51**	**F**	**III**	**NFPA**	+
**23**	**45**	**F**	**IV**	**NFPA**	+
**24**	**31**	**F**	**IV**	**NFPA**	+
**25**	**59**	**M**	**III**	**GH**	+
**26**	**42**	**F**	**IV**	**GH**	+
**27**	**31**	**M**	**IV**	**PRL**	**-**
**28**	**57**	**M**	**III**	**NFPA**	**-**
**29**	**37**	**M**	**III**	**PRL**	**-**
**30**	**43**	**F**	**III**	**PRL**	+
**31**	**16**	**M**	**III**	**NFPA**	+
**32**	**63**	**M**	**III**	**NFPA**	**-**
**33**	**28**	**F**	**III**	**GH**	+
**34**	**59**	**F**	**III**	**NFPA**	+
**35**	**55**	**M**	**IV**	**NFPA**	+
**36**	**60**	**F**	**III**	**NFPA**	+
**37**	**29**	**F**	**IV**	**NFPA**	+
**38**	**46**	**F**	**III**	**GH**	**-**
**39**	**30**	**M**	**IV**	**GH**	+
**40**	**46**	**F**	**IV**	**GH**	+

F, female; M, male; PRL, Prolactin; GH, growth hormone; NFPA, nonfunctioning pituitary adenoma; "+" indicates Ki-67 index≥3%; "-" indicates Ki-67 index <3%.

Supplementary Table S2. Four invasive PA patients

Case	Age	Gender	Tumor size(cm)	Knosp grade	Hormonal type
1	51	F	2.2x2.4x3.3	IV	GH
2	66	F	2.6x2.7x2.0	III	GH
3	78	M	3.6x1.5x2.5	IV	NFPA

4	43	M	2.8x2.9x2.1	IV	NFPA

M: male; F: female; GH, growth hormone; NFPA, nonfunctioning pituitary adenoma

Supplementary Table S3. Primer list for qPCR

Gene name (Species)	Primer sequences (5'-3')	Tm cycles	Product (bp)
LDHA(human)	F: GGTTGGTGCTGTTGGCATGG	58-35	214
	R: TGCCCCAGCCGTGATAATGA		
β-actin (human)	F: GCACCACACCTTCTACAATGAGC	58-35	163
	R: TAGCACAGCCTGGATAGCAACG		
LDHA(rat)	F: CAAACTGCTCATCGTCTCAAACC	57-30	129
	R: ATCAGGTAACGGAACCGAGCC		
β-actin (rat)	F: GAGGGAAATCGTGCGTGAC	57-30	157
	R: GCATCGGAACCGCTCATT		
